# A Validation Study of the Web Screening Questionnaire (WSQ) Compared With the Mini-International Neuropsychiatric Interview-Plus (MINI-Plus)

**DOI:** 10.2196/mental.5453

**Published:** 2017-08-29

**Authors:** Denise Meuldijk, Erik J Giltay, Ingrid VE Carlier, Irene M van Vliet, Albert M van Hemert, Frans G Zitman

**Affiliations:** ^1^ School of Psychology Illawarra Health and Medical Research Institute University of Wollongong Wollongong, New South Wales Australia; ^2^ Department of Psychiatry Leiden University Medical Centre Leiden Netherlands

**Keywords:** depressive disorders, anxiety disorders, surveys and questionnaires, diagnostic, brief, clinical practice

## Abstract

**Background:**

There is a need for brief screening methods for psychiatric disorders in clinical practice. This study assesses the validity and accuracy of a brief self-report screening questionnaire, the Web Screening Questionnaire (WSQ), in detecting psychiatric disorders in a study group comprising the general population and psychiatric outpatients aged 18 years and older.

**Objective:**

The aim of this study was to investigate whether the WSQ is an adequate test to screen for the presence of depressive and anxiety disorders in clinical practice.

**Methods:**

Participants were 1292 adults (1117 subjects from the general population and 175 psychiatric outpatients), aged 18 to 65 years. The discriminant characteristics of the WSQ were examined in relation to the (“gold standard”) Mini-International Neuropsychiatric Interview-Plus (MINI-Plus) disorders, by means of sensitivity, specificity, area under the curve (AUC), and positive and negative predictive values (PPVs, NPVs).

**Results:**

The specificity of the WSQ to individually detect depressive disorders, anxiety disorders, and alcohol abuse or dependence ranged from 0.89 to 0.97 for most disorders, with the exception of post-traumatic stress disorder (0.52) and specific phobia (0.73). The sensitivity values ranged from 0.67 to 1.00, with the exception of depressive disorder (0.56) and alcohol abuse or dependence (0.56). Given the low prevalence of separate disorders in the general population sample, NPVs were extremely high across disorders (≥0.97), whereas PPVs were of poor strength (range 0.02-0.33).

**Conclusions:**

In this study group, the WSQ was a relatively good screening tool to identify individuals without a depressive or anxiety disorder, as it accurately identified those unlikely to suffer from these disorders (except for post-traumatic stress disorders and specific phobias). However, in case of a positive WSQ screening result, further diagnostic procedures are required.

## Introduction

Structured diagnostic interviews such as the Composite International Diagnostic Interview (CIDI) [1] and the Structured Clinical Interview for DSM-III-R (SCID) [2] are considered gold standards in research, used to diagnose psychiatric disorders in a standardized way [1,3,4]. However, they are less suitable for clinical practice because their administration is time consuming, and they can only be administered by well-trained interviewers [5]. The Mini-International Neuropsychiatric Interview-Plus (MINI-Plus) [6] is a much shorter diagnostic interview with diagnostic properties similar to the CIDI [6,7]. However, the MINI-Plus also requires trained interviewers and takes up to 30 min to complete, making it costly for routine use in clinical practice. Therefore, because these interviews are often impractical to be used as a screener for routine use, a reliable, valid, and briefly self-rating screening questionnaire is desired. The Web Screening Questionnaire (WSQ) [8] was developed to quickly screen for common psychiatric disorders (ie, anxiety or depressive disorders and alcohol abuse or dependence). This Internet-based, self-report screening questionnaire consists of only 15 items and requires less than 5 min to complete. The WSQ has good to excellent validity for social phobia, panic disorder with agoraphobia, agoraphobia (without panic disorder), obsessive compulsive disorder (OCD), and alcohol abuse or dependence (sensitivity ranges from 0.72-1.00; and specificity from 0.63-0.80) [8]. Slightly more modest psychometric properties were reported for depressive disorder, generalized anxiety disorder (GAD), post-traumatic stress disorder (PTSD), specific phobia, and panic disorder (without agoraphobia), that is, sensitivity 0.80 to 0.93; specificity 0.44 to 0.51 [8]. These data reflect the validation of the WSQ compared with CIDI diagnoses ascertained in the general population with 6-month prevalence rates of the Diagnostic and Statistical Manual, 4th edition-Text Revision (DSM-IV-TR) diagnoses [9]. As the WSQ screens for current symptoms [8], it is relevant to test the WSQ against current DSM-IV diagnoses.

This study examines the validity and accuracy of the WSQ as a screener against 1-month prevalence MINI-Plus disorders covered by the WSQ. The study group mainly comprised a general population sample recruited from primary care registrations. To increase the prevalence of psychiatric disorders, we enriched this general population sample with a smaller sample of psychiatric outpatients to form one large study group.

## Methods

### Sample

For this study, to ensure statistical power of the analyses, participants from a general population study and participants from a pragmatic randomized controlled trial (RCT) conducted in clinical practice were combined into one large study group.

The 1302 participants from the general population were recruited (from November 2009 to January 2011) from the administration of eight university-affiliated general practices in the vicinity of Leiden, the Netherlands. In the Netherlands, since nearly 100% of the population is registered with a general practitioner (GP), the primary care sample is equivalent to a general population sample [10,11]. To form a nonpatient control group, representative of a population referred for suspected (but not necessarily diagnosed with) mood, anxiety and/or somatoform disorders, four exclusion criteria were applied by Schulte-van Maaren and colleagues (2013) [12]: (1) treatment in a secondary psychiatric care center in the last 6 months for psychiatric problems and/or dependence on alcohol or drugs; (2) hearing impairment or limited cognitive abilities such as aphasia, severe dyslexia, or dementia; (3) illiteracy or insufficient mastery of the Dutch language; and (4) suffering from a potentially lethal disorder. The initial study was designed to generate reference values in primary care for questionnaires used in the assessment of psychopathology. Details of this study by Schulte-van Maaren and colleagues (2013) are described elsewhere [12]. This study focuses on the main aspects relevant for the current research question.

The general population sample derived from the study of Schulte-van Maaren et al (2013) [12] was enriched with a sample of 182 secondary care outpatients who were originally recruited for a pragmatic RCT and in whom the WSQ and the MINI-Plus were assessed at baseline. This RCT is published in Meuldijk and colleagues (2012) [13]. The trial was conducted (from March 2010 to December 2012) at five outpatient mental health clinics in and around Leiden of Rivierduinen (RD), a secondary Regional Mental Health Provider (RHMP) in the province of South-Holland, the Netherlands. Eligible participants were patients aged 18-65 years, referred to the mental health clinics by their GP for the treatment of a current mild to moderate anxiety and/or depressive disorder including depressive disorder, dysthymia, panic disorder (with or without agoraphobia), social phobia, specific phobia, GAD, OCD, and PTSD. Exclusion criteria were (1) suicidal or homicidal risk; (2) delusions, hallucinations, bipolar, or psychotic disorder; (3) severe social dysfunction; and/or (4) insufficient knowledge of the Dutch language.

In both subsamples, the assessment included (among others) the MINI-Plus and the WSQ. Of the initial general population sample of 1302 participants, 185 had incomplete WSQ data, leaving 1117 participants for inclusion in the present analysis. Of the outpatient sample of 182 patients, 6 had incomplete WSQ data and 1 MINI-Plus interview was incomplete, resulting in 175 outpatients. Thus, the (combined) study group for this study consisted of (1117+175) 1292 participants.

The study protocol for both samples was approved by the medical ethical committee of the Leiden University Medical Center.

### Web Screening Questionnaire (WSQ)

The WSQ (see Multimedia Appendix 1) is a 15-item, self-report instrument that screens for depressive disorder, GAD, panic disorder with or without agoraphobia, social phobia, specific phobia, OCD, PTSD, agoraphobia, suicidality, and alcohol abuse or dependence [8]. The RCT of Meuldijk and colleagues excluded participants with a moderate to high suicidality risk and/or suicidal ideation [13]. Therefore, in this study, the WSQ item that assesses the risk of suicide or self-harm was not included in the analysis. The WSQ is based on the screening questionnaire of Marks and colleagues [14]. Compared with the 6-months CIDI diagnoses, in the general population, the WSQ has moderate to good screening properties (sensitivity 0.72 to 1.00; specificity 0.44 to 0.80) [8]. Depression, panic disorder with agoraphobia, and alcohol dependence were each assessed by two items, whereas the other disorders were assessed by single items. The same WSQ cut-off scores were applied as used in the study by Donker and colleagues (2006) [8].

### Mini-International Neuropsychiatric Interview-Plus (MINI-Plus)

The MINI-Plus 5.0.0, Dutch version, was used as the “gold standard” reference [6]. The MINI-Plus is a structured and standardized diagnostic interview used to determine the most common psychiatric disorders according to axis I DSM-IV-TR [9] and the International Classification of Diseases and Related Health Problems (ICD-10) [6].

For this study, we used the diagnoses of (1) mood disorders (depression and dysthymia), (2) anxiety disorders (panic disorder with or without agoraphobia, agoraphobia, social phobia, specific phobia, GAD, PTSD [type I or single trauma], and OCD), and (3) alcohol abuse or dependence. The MINI-Plus has good psychometric properties and is widely used to support diagnostics in psychiatry. The MINI-Plus was conducted by trained research nurses. As the WSQ screens for current diagnoses, only the 1-month MINI-Plus was used.

### Statistical Analyses

The discriminant function of the WSQ was assessed for each of the MINI-Plus Axis 1 DSM-IV-TR disorders for which it screens, using sensitivity, specificity, receiver operating characteristics (ROC) curve (area under the curve [AUC]) [15], and positive and negative predictive values (PPVs, NPVs). Specificity was calculated as the proportion of patients who did not have the MINI-plus diagnosis and who had a negative WSQ screen. Sensitivity was determined as the proportion of patients with a MINI-Plus psychiatric diagnosis who had a positive WSQ screen for the same disorder. The AUC, (interpreted as the probability that a randomly selected clinical case will score higher on the test than a noncase), is not sensitive to prevalence and is proposed to correct this problem [16]; it can range from 0.50 (worthless test) to 1.00 (perfect test). Following Agresti (2002) [17], we considered the AUC to be of excellent evidence of concordance if ≥0.90, good evidence of concordance if between 0.80 and 0.90, acceptable although only average if between 0.70 and 0.80, and poor if <0.70. The PPV was calculated as the percentage of participants with a positive test on the WSQ who actually had the disorder according to the MINI-Plus diagnosis, whereas the NPV was calculated as the percentage of participants with a negative test that did not have the disorder according to the MINI-Plus. As the PPV and the NPV strongly depend on the prevalence of the disorder, we calculated these indices on the general population sample only, without the enrichment; otherwise, the results would be artificially inflated. Furthermore, WSQ cut-off scores were applied as originally recommended by Donker et al 2006 [8] and slightly adapted to fit within routine outcome monitoring (ROM), a monitoring system for psychiatric patient care [18]. All analyses were conducted using IBM SPSS version 20.0 for Windows.

## Results

### Demographics and Prevalence of Diagnostic and Statistical Manual, 4th Edition-Text Revision (DSM-IV-TR) Diagnoses

Characteristics of the two subsamples are presented in Table 1. In the total study group, the mean age was 39.6 years (range 18-65, standard deviation [SD]=12.6), and 60.53% (782/1292) of the participants were female. Most participants were of Dutch origin (1223/1292; 94.66%) and had completed a higher level of education (972/1292; 75.23%). At baseline, 77.32% of the participants (999/1292) were employed, and 66.18% (855/1292) were married. In the total group, 79 participants (6.11%) met the DSM-IV-TR MINI-Plus criteria for a current (ie, within the past month) depressive (with or without anxiety) disorder. Of the total group, 139 participants (10.76%) met the criteria for an anxiety with or without a depressive disorder; these participants were diagnosed according to the common subtypes of anxiety as indicated in Table 2. In addition, 55 participants (4.26%) met the criteria for current alcohol abuse or dependence disorder. The majority of the study group (934/1292, 72.29%) did not pass the threshold for a current MINI-Plus diagnosis. It is recognized that the two study groups are not the same. The study population contains selected subgroups of particular interest; the difference in clinical and demographic characteristics within these subgroups contributes to define the target population.

**Table 1:** Baseline sociodemographic and clinical characteristics of the two subsamples and the total study group (n=1292). The MINI International Neuropsychiatric Interview-Plus (MINI-Plus) 5.0.0 was used to collect diagnostic information. Participants can have more than one diagnosis.

Characteristics			General population sample (n=1117)	Outpatient sample (n=175)	Total study sample (n=1292)
**Baseline sociodemographic^a^** **characteristics**						
	Age (years), mean (SD^b^)		40.04 (12.53)	36.67 (12.40)	39.6 (12.56)
	**Gender, n (%)**					
		Female	712 (63.74)	70 (40.0)	782 (60.53)
	**Ethnical background^c^, n (%)**					
		Dutch	1116 (99.91)	160 (91.4)	1223 (94.66)
		Other	53 (4.74)	10 (5.7)	63 (4.88)
	**Educational status^d^, n (%)**				
		Lower education	250 (22.38)	64 (36.6)	314 (24.30)
		Higher education	866 (77.53)	106 (60.6)	972 (75.23)
	**Employment status, n (%)**				
		Employed	914 (81.83)	85 (48.6)	999 (77.32)
		Unemployed or retired	202 (18.08)	85 (48.6)	287 (22.21)
	**Marital status, n (%)**				
		Married or cohabitating	766 (68.58)	89 (50.9)	855 (66.18)
**Clinical characteristics or MINI^e^-Plus Diagnosis^f^, n (%)**						
	Depressive (with or without anxiety) disorder		12 (1.07)	67 (38.3)	79 (6.11)
	Anxiety (with or without depressive) disorder		60 (5.37)	79 (45.1)	139 (10.76)
	Panic disorder (without agoraphobia)		4 (0.36)	24 (13.7)	28 (2.17)
	Agoraphobia		27 (2.42)	37 (21.1)	64 (4.95)
	Panic disorder with agoraphobia		2 (0.18)	18 (10.3)	20 (1.55
	Social phobia		10 (0.09)	9 (5.1)	19 (1.47)
	Specific phobia		9 (0.81)	3 (1.7)	12 (0.93)
	Generalized anxiety disorder		13 (1.16)	22 (12.6)	35 (2.71)
	Posttraumatic stress disorder		5 (0.45)	14 (8.0)	19 (1.47)
	Obsessive compulsive disorder		6 (0.54)	3 (1.7)	9 (0.70)
	Alcohol abuse or dependence		51 (4.57)	4 (2.3)	55 (4.26)
	No current DSM-IV-TR^g^ diagnosis^h^		902 (80.75)	32 (18.3)	934 (72.29)

^a^Demographic data; ethnic background, educational status, and employment status are missing for 6 participants (1 participant from the general population sample, and 5 outpatients).

^b^SD: standard deviation.

^c^Dutch ethnic background was assumed when the participant was born in the Netherlands.

^d^Lower education=having completed elementary school, lower general primary education, or no education at all; higher education=more than lower education (includes university).

^e^MINI: Mini-International Neuropsychiatric Interview.

^f^Clinical characteristics or diagnosis were missing for 1 participant.

^g^DSM-IV-TR: Diagnostic and Statistical Manual of Mental Disorders, 4th edition-Text Revision.

^h^Denotes participants who did not pass the threshold for having a current Axis- I DSM-IV-TR diagnosis according to the MINI-Plus interview.

**Table 2:** Agreement between the Mini-International Neuropsychiatric Interview (MINI)-Plus and the Web Screening Questionnaire (WSQ) for individual disorders in the total sample (n=1292). Numbers in the table reflect the use of each screening subscale to detect any diagnosis rather than only the diagnosis associated with the subscale. WSQ cut-off scores were derived from the original cut-offs recommended by Donker et al (2009) [8]. WSQ cut-off scores: depression: Q1≥5 and Q2=1; panic disorder: Q4≥1; agoraphobia Q5=1; panic disorder with agoraphobia Q4≥1 and Q5=1; social phobia: Q8=1 and Q9=1; specific phobia: Q6 or Q7=1; generalized anxiety disorder (GAD): Q3≥2; post-traumatic stress disorder (PTSD): Q10=1 or Q11=1; obsessive compulsive disorder (OCD): Q12≥1; and alcohol abuse or dependence: Q13≥2 and Q14≥3.

DSM-IV-TR^a^ diagnosis	MINI^b^ prevalence (%)	WSQ^c^ prevalence (%)	True positive	False positive	False negative	True negative	Specificity (95% CI)	Sensitivity (95% CI)	AUC^d^ (95% CI)
Depressive disorder	79 (6.11)	115 (8.90)	46	69	33	1144	0.94 (0.93-0.96)	0.58 (0.47-0.69)	0.83 (0.68-0.98)
Panic disorder	28 (2.16)	170 (13.16)	28	142	0	1122	0.89 (0.87-0.90)	1.00 (0.88-1.00)	0.98 (0.96-1.00)
Agoraphobia	64 (4.95)	111 (8.59)	52	59	12	1169	0.95 (0.94-0.96)	0.81 (0.70-0.90)	0.80 (0.69-0.91)
Panic disorder with agoraphobia	20 (1.55)	61 (4.72)	18	43	2	1229	0.97 (0.96-0.98)	0.90 (0.68-0.99)	0.99 (0.98-1.00)
Social phobia	19 (1.47)	101 (7.82)	15	86	4	1187	0.93 (0.92-0.95)	0.79 (0.54-0.94)	0.95 (0.92-0.99)
Specific phobia	12 (0.93)	363 (28.10)	12	351	0	929	0.73 (0.70-0.75)	1.00 (0.74-1.00)	0.93 (0.89-0.97)
Generalized anxiety disorder	35 (2.71)	145 (11.22)	23	122	12	1135	0.90 (0.89-0.92)	0.66 (0.48-0.81)	0.89 (0.79-0.99)
Post-traumatic stress disorder	19 (1.47)	621 (48.07)	15	606	4	667	0.52 (0.50-0.55)	0.79 (0.54-0.94)	0.86 (0.74-0.98)
Obsessive compulsive disorder	9 (0.69)	120 (9.3)	6	114	3	1169	0.91 (0.89-0.92)	0.67 (0.30-0.93)	0.82 (0.59-1.00)
Alcohol abuse or dependence	55 (4.26)	121 (9.37)	31	90	24	1147	0.93 (0.91-0.94)	0.56 (0.42-0.70)	0.82 (0.75-0.88)

^a^DSM- IV-TR: Diagnostic and Statistical Manual of Mental Disorders, 4th edition-Text Revision.

^b^MINI: Mini International Neuropsychiatric Interview; MINI-Plus 5.0.0.

^c^WSQ: Web Screening Questionnaire.

^d^AUC: area under the curve.

### Concordance Between Mini-International Neuropsychiatry Interview (MINI)-Plus and Web Screening Questionnaire (WSQ)

**Figure 1:**
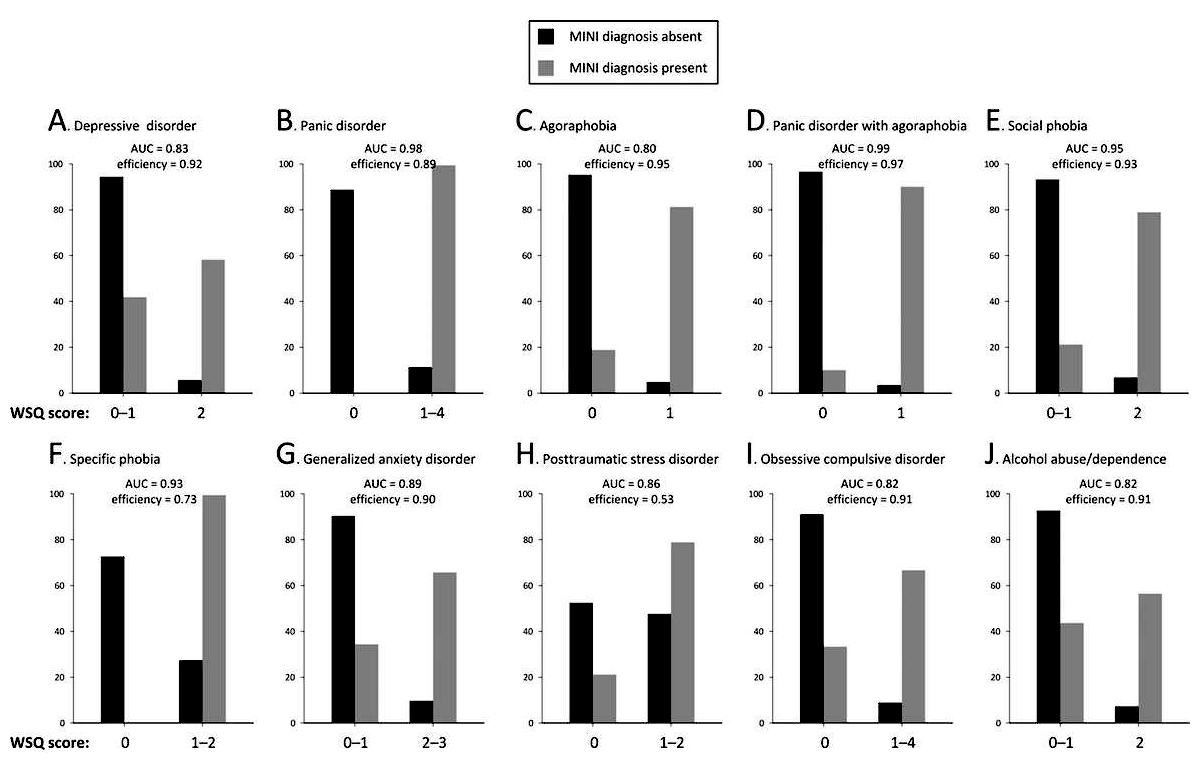
Distribution of the MINI-Plus diagnosis for the corresponding Web Screening Questionaire (WSQ) subscales in the study sample (N = 1292) MINI-Plus=The MINI International Neuropsychiatric Interview-Plus 5.0.0. WSQ=Web Screening Questionnaire. WSQ cut-off scores were derived from the original cut-offs recommended by Donker and colleagues (2009) [8]. WSQ cut-off scores: depression: Q1≥5 and Q2=1; panic disorder: Q4 ≥1; agoraphobia: Q5=1; panic disorder with agoraphobia Q4≥1 and Q5=1; social phobia: Q8=1 and Q9=1; specific phobia: Q6 or Q7=1; generalized anxiety disorder (GAD): Q3≥2; post-traumatic stress disorder (PTSD): Q10=1 or Q11=1; obsessive compulsive disorder (OCD): Q12≥1; and alcohol abuse or dependence : Q13≥2 & Q14≥3. MINI-Plus: The Mini-International Neuropsychiatric Interview-Plus 5.0.0. WSQ: Web Screening Questionnaire.

The concordance between each diagnosis classified according to the DSM-IV-TR with the MINI-Plus and the WSQ questionnaire is presented in Table 2. Specificity was high (range 0.89-0.97) for most individual disorders, with the exception of specific phobia (0.73) and PTSD (0.52). Sensitivity was substantial to high (0.67 to 1.00) for the majority of disorders. The exceptions were depressive disorder (0.58) and alcohol abuse or dependence (0.56). All AUC values were good to excellent (≥0.82) for the individual disorders. The best discriminating subscale was panic disorder with agoraphobia (AUC=0.99), followed by panic disorder (AUC=0.98) and social phobia (AUC=0.95). Figure 1 presents the discriminative power of each subscale of the WSQ. Data on PPCs and NPVs are given in Table 3. These indices were calculated for the general population subsample only because of the strong relation to the prevalence of the disorders. Despite generally strong discriminative power, the PPV was of poor strength ranging from 0.01 (PTSD) to 0.33 (agoraphobia); the NPVs were uniformly high (≥0.97) for all scales.

**Table 3:** Predictive value of the Web Screening Questionnaire (WSQ) for individual disorders according to the Mini-International Neuropsychiatry Interview (MINI)-Plus in the general population subsample (n=1117). Numbers in the table reflect the use of each screening subscale to detect any diagnosis rather than only the diagnosis associated with the subscale. WSQ cut-off scores were derived from the original cut-offs recommended by Donker et al (2009) [8]. WSQ cut-off scores: depression: Q1≥5 and Q2=1; panic disorder: Q4≥1; agoraphobia: Q5=1; panic disorder with agoraphobia Q4≥1 and Q5=1; social phobia: Q8=1 and Q9=1; specific phobia: Q6 or Q7=1; generalized anxiety disorder (GAD): Q3≥2; post-traumatic stress disorder (PTSD): Q10=1 or Q11=1; obsessive compulsive disorder (OCD): Q12≥1; and alcohol abuse or dependence: Q13≥2 and Q14≥3.

DSM-IV-TR^a^ diagnosis	MINI^b^ prevalence (%)	WSQ^c^ prevalence (%)	True positive	False positive	False negative	True negative	PPV^d^ (95% CI)	NPV^e^ (95% CI)
Depressive disorder	12 (1.107	28 (2.51)	6	22	6	1083	0.21 (0.08-0.41)	0.99 (0.99-1.00)
Panic disorder	4 (0.36)	64 (5.73)	4	60	0	1053	0.06 (0.02-0.15)	1.00 (0.99-1.00)
Agoraphobia	27 (2.42)	52 (4.66)	17	35	10	1055	0.33 (0.20-0.47)	0.99 (0.98-1.00)
Panic disorder with agoraphobia	2 (0.18)	11 (0.98)	2	9	0	1106	0.18 (0.02-0.52)	1.00 (0.99-1.00)
Social phobia	9 (0.81)	47 (4.21)	7	40	3	1067	0.15 (0.06-0.28)	1.00 (0.99-1.00)
Specific phobia	9 (0.81)	281 (25.16)	9	272	0	836	0.03 (0.01-0.06)	1.00 (0.99-1.00)
Generalized anxiety disorder	13 (1.16)	46 (4.12)	8	38	5	1066	0.17 (0.08-0.31)	1.00 (0.99-1.00)
Post-traumatic stress disorder	5 (0.45)	511 (45.75)	5	506	0	606	0.01 (0.03-0.02)	1.00 (0.99-1.00)
Obsessive compulsive disorder	6 (0.54)	55 (4.92)	4	51	2	1060	0.07 (0.02-0.18)	1.00 (0.99-1.00)
Alcohol abuse or dependence	51 (4.57)	110 (9.85)	28	82	23	984	0.25 (0.18-0.35)	0.98 (0.97-0.99)

^a^DSM-IV-TR: Diagnostic and Statistical Manual of Mental Disorders, 4th edition-Text Revision.

^b^MINI: Mini International Neuropsychiatric Interview; MINI-Plus 5.0.0.

^c^WSQ: Web Screening Questionnaire.

^d^PPV: positive predictive value.

^e^NPV: negative predictive value.

## Discussion

### Principal Findings

This study evaluated the feasibility of the WSQ to screen for DSM-IV-TR diagnoses of depressive disorder, anxiety disorders, and alcohol abuse or dependence. Overall, the WSQ was relatively successful in discriminating between individuals with and without a MINI-Plus diagnosis. However, if the WSQ tests positive for a psychiatric disorder, further examination is warranted because of the poor PPVs. Thus, most patients who tested positively, did not receive a MINI-Plus diagnosis.

The adequate strength of the findings regarding sensitivity, specificity, and AUC values suggest that the WSQ has some desirable screening characteristics. Its high sensitivity suggests that it may help to confirm the absence of most of these psychiatric diagnoses, that is, ruling out the disorders. However, the exceptions are depressive disorder, specific phobia, PTSD, and alcohol abuse or dependence, for which the agreement in ruling out these psychiatric disorders was lower. In the general population subsample, the NPVs were high, but the PPVs were relatively low compared with the MINI-Plus results. Although the PPVs and NPVs are not intrinsic to the test, they are directly related to the prevalence of the disease in the population. Assuming all other factors remain constant, PPV increases with increasing prevalence, and NPV decreases with an increase in prevalence.

Together with the results reported by Donker and colleagues [8] who found AUCs of 0.65 to 0.83 in their validation of the WSQ against DSM-IV-TR diagnoses with the CIDI in the general population, the present results indicate that the WSQ has potential as a screener, ruling out the presence of a disorder.

Our findings are in line with other validation studies comparing brief screening tools with longer ones and also showing the feasibility of these short screening instruments. This applies, for example, to the Psychiatric Diagnostic Screening Questionnaire (PDSQ) in outpatients [19,20] and the Mental Health Inventory 5 (MHI-5) and the Anxiety and Depression Detector (ADD) for primary care populations [21-23].

### Strengths and Limitations

A strength of this study is the large number of participants included. Another strength is that our MINI-Plus data allowed to determine the concordance of the WSQ with the last 1-month DSM-IV-TR diagnoses, providing an accurate measure of the current (or very recent) prevalence of this mental disorder. In contrast, Donker and colleagues (2009) [8] used 6-month prevalence rates, which implies that some disorders could have receded during the past 5 months.

A limitation of this study is that all the GP practices included were affiliated with a university hospital. Because such practices tend to have more focus on research and training than nonaffiliated practices, this may have introduced bias in the study group population. Moreover, the study group was mainly compiled from participants included in an earlier general population study [12]; these participants did not have psychiatric treatment for 6 months before recruitment and did not report dependence on alcohol or other drugs. Although we made considerable effort to compensate for this potential source of bias in recruitment by adding a psychiatric outpatient subsample [13], the prevalence of psychiatric disorders in our general population subsample was substantially lower than expected from population-based surveys in the Netherlands [24,25]. Therefore, it is likely that we probably included an overly healthy study group, thereby limiting the generalizability of these results to the general population or other patient samples. Therefore, the present results need to be confirmed in other study populations. As a result, the NPV estimates may have been too high and the PPV too low. Our findings with regard to the predictive value must be considered extreme, given the very low prevalence of disorders in the present sample. In addition, as the number of participants with certain conditions was small, this yielded less precise effect estimates, which should be taken into account when interpreting these results. Predictive values from one study should not be transferred to another setting with a different prevalence of the disease in the population. However, our estimates of the sensitivity, specificity, and AUC are not affected by this limitation. Moreover, reconsidering the diagnostic criteria and the screening items used for the individual WSQ items could contribute to a higher accuracy of detecting disorders and higher positive and predictive values.

A final limitation is that our sample was restricted to Dutch-speaking individuals able to write (illiterate or non-Dutch speaking persons were excluded); this may also limit the generalizability of our results, especially across different immigrant groups. In addition, future research could investigate the impact of demographic factors on our study results. Although it is generally assumed that structured diagnostic interviews (ie, the MINI-Plus and SCID) are the “gold standards” for the assessment of diagnoses in psychiatric research, these standards have their limitations. One advantage for reproducibility is that it is fully clear what standardized procedures had been followed. However, the notion that mental disorders (eg, depression and anxiety) are entities that can be diagnosed remains debatable, despite the apparent clinical value of such diagnoses.

### Conclusions

The WSQ is a short questionnaire to screen for depression, GAD, panic disorder with or without agoraphobia, social phobia, specific phobia, OCD, PTSD, and alcohol abuse or dependence. In has proven useful in the general population to screen for the 6-months prevalence for these disorders, compared with the CIDI as gold standard [8]. This study yielded similar results, with the 1-month prevalence of these disorders in the MINI-Plus in a general practice population, combined with psychiatric outpatients. Taken together, exploring the agreement between both instruments, or findings, indicates that the WSQ can (potentially) be used as a brief and less costly screening tool for depressive or anxiety disorders (except for PTSD and specific phobia). The WSQ seems a promising tool with a two-step diagnostic approach, for example, in primary care. It could assist health care providers in screening patients before consultation. Patients who screen positive should undergo more extensive diagnostic procedures, whereas a negative screen indicates that it is highly unlikely that further evaluation would be useful. With such an approach, diagnostic accuracy might be increased and costly diagnostic procedures limited or avoided.

However, this study had several limitations which should be considered when interpreting and generalizing our finding to other groups. For example, participant recruitment was not standard, and recruitment barriers could not be completely eliminated. Also, in our study group, the prevalence of psychiatric disorders was lower than expected. Moreover, concern still exists about the usefulness of the WSQ in its current form; some revision of the scale may be required to improve its psychometric properties. Future research exploring the feasibility of the WSQ for assessing mental health in general practice might be a further step in the economization and optimization of mental health care.

## Appendix

Multimedia Appendix 1 Web Screening Questionnaire (WSQ).

## Abbreviations

ADD: anxiety and depression detector
AUC: area under the curve
CIDI: Composite International Diagnostic Interview
DSM-IV-TR: Diagnostic and Statistical Manual of Mental Disorders, 4th edition-Text Revision
GP: general practitioner
ICD-10: The International Classification of Diseases and Related Health Problems
GAD: generalized anxiety disorder
LUMC: Leiden University Medical Center
MEC: Medical Ethical Committee
MHI-5: Mental Health Inventory-5
MHC: mental health clinics
MINI-Plus: Mini-International Neuropsychiatric Interview-Plus
NPV: negative predictive values
OCD: obsessive compulsive disorder
PPV: positive predictive values
PDSQ: Psychiatric Diagnostic Screening Questionnaire
PTSD: post-traumatic stress disorder
RCT: randomized controlled trial
RD: Rivierduinen
RHMP: regional mental health provider
ROC: receiver operating characteristic
ROM: routine outcome monitoring
WSQ: Web Screening Questionnaire
